# Neonatal Exposure to Amoxicillin Alters Long-Term Immune Response Despite Transient Effects on Gut-Microbiota in Piglets

**DOI:** 10.3389/fimmu.2019.02059

**Published:** 2019-09-04

**Authors:** Janelle M. Fouhse, Kaiyuan Yang, Juan More-Bayona, Yanhua Gao, Susan Goruk, Graham Plastow, Catherine J. Field, Daniel R. Barreda, Benjamin P. Willing

**Affiliations:** ^1^Department of Agricultural, Food and Nutritional Science, University of Alberta, Edmonton, AB, Canada; ^2^Department of Biological Sciences, University of Alberta, Edmonton, AB, Canada; ^3^College of Life Science and Technology, Southwest Minzu University, Chengdu, China

**Keywords:** antibiotic, microbiota, immune development, *Salmonella*, pig model

## Abstract

Antibiotic exposure during neonatal development may result in transient or persistent alterations of key microbes that are vital for normal development of local and systemic immunity, potentially impairing immune competence later in life. To further elucidate the relationship between antibiotic exposure and immune development, newborn pigs were exposed to a therapeutic pediatric dose (30 mg/kg/day) of amoxicillin (AB) or placebo (PL) from post-natal day (PND) 0–14. Subsequently, immune cell phenotype, microbial composition, and immune response to an intraperitoneal (IP) challenge with *Salmonella enterica* serovar Typhimurium were evaluated. AB exposure caused significant changes in fecal microbial composition on PND 3 (*P* = 0.025). This stemmed from a 2-fold increase in *Enterobacteriaceae* with live cecal coliforms on PND 7 indicating at 10-fold increase (*P* = 0.036). Alterations in microbial composition were transient, and successional patterns were normalizing by PND 14 (*P* = 0.693). Differences in PBMC (peripheral blood mononuclear cell) immune cell subtypes were detected, with the percentage of CD3^+^CD4^+^ T cells among the broader T cell population (CD3^+^CD4^+^/CD3^+^) being significantly higher (*P* = 0.031) in AB pigs and the numbers of CD4+CD45RA+ (naïve) T cells per liter of blood were lower on PND 21 in AB pigs (*P* = 0.036). Meanwhile, PBMCs from AB pigs produced significantly more IFNγ upon stimulation with a T-cell mitogen on PND 21 and 49 (*P* = 0.021). When AB pigs were challenged with heat-killed *Salmonella* (IP) on PND 49, IFNγ gene expression in peripheral blood was upregulated compared to those treated with PL (*P* = 0.043). Additionally, AB pigs showed stronger activation among neutrophils infiltrating the peritoneal cavity after *in vivo* immune challenge, based on higher levels of NF-κB nuclear translocation (*P* = 0.001). Overall, our results indicate that early life treatment with a therapeutically relevant dose of a commonly prescribed antibiotic has a programming effect on the immune system. Despite antibiotics only causing a transient disruption in gut-associated microbial communities, implications were long-term, with antibiotic treated pigs mounting an upregulated response to an immune challenge. This research adds to the growing body of evidence indicating adverse immune outcomes of early life antibiotic exposures.

## Introduction

Antibiotics are life-saving medications used to treat bacterial infections. However, they are frequently incorrectly or over prescribed (1, 2). Infants and young children are more vulnerable to infections as their immune system is not fully developed (3), and are prescribed antibiotics at much higher rates in comparison to adults (4–6). Children between the ages 0–2 have the highest rate of antibiotic prescriptions in comparison to older children and adults in US, Germany, and other European countries (7–9). Approximately 69% of children are exposed to antibiotics within 2 years of age in the United States and 45% of infants in Canada during the periparturient period (10, 11).

Antibiotic exposure in infants has been linked to higher risk of developing disorders with immune involvement later in life, such as obesity, diabetes, asthma, inflammatory bowel disease, among others (12–14). Antibiotics may cause transient or persistent alterations in gut-associated microbiota and are suggested to be a major contributor to increased prevalence of immune-mediated disorders (15). Post-natal microbial colonization in the gastrointestinal tract greatly influences the development of an infant's immune system (16, 17). A critical period of time during the early developmental period has been proposed during which dysbiosis impedes immune system development, potentially increasing host susceptibility to immune disorders including both autoimmune conditions (18) and immune-compromised conditions (15).

In non-obese diabetic mice, continuous vancomycin treatment (0.2 mg/mL) from conception led to a partial loss of the gut microbiota, changed T cell subsets and significantly increased type 1 diabetes incidence (19). Exposing mice to vancomycin *in utero* and post-birth altered colonic T-regulatory cells and increased the severity of allergic asthma (20). In addition, peripartum exposure to cefoperazone caused persistent gut dysbiosis and aberrant development of the offspring immune system, which increased the disease risk in spontaneous and chemically-induced colitis mouse models (21). These studies support the role of antibiotic-induced dysbiosis leading to increased susceptibility to immune mediated disorders later in life. However, very few studies have examined clinically relevant antibiotics at therapeutic doses in robust animal models.

Pigs provide a clinically relevant model to study human disease due to their comparable size and similar anatomical and immune features to human infants including being immunologically naïve at birth (22, 23). Previously, our lab has established a swine model using post-natal amoxicillin exposure including a transient shift in gut microbiota composition with persisting metabolic defects (24). In the present study, we implemented the same animal model to investigate the effects of an early-life standard therapeutic dose of amoxicillin on host immune function in order to determine if microbial alterations in early life disrupt immune competence later in life. We hypothesized that neonatal antibiotic exposure of pigs would lead to a disrupted fecal microbial successional pattern, and modify immune cell phenotype and function, altering immune responsiveness when challenged with an intraperitoneal (IP) injection of heat-killed *Salmonella* later in life. Immune cell (PBMC) function was assessed by *ex vivo* cytokine production, and immune responsiveness was measured post IP challenge by evaluating leukocyte NF-κB nuclear translocation and ROS production.

## Materials and Methods

### Animal Maintenance

The animal study was approved by the Animal Care and Use Committee of the University of Alberta and conducted in accordance to the guidelines of the Canadian Council on Animal Care at the Swine Research and Technology Centre (Edmonton, AB, Canada). A total of 84 crossbred pig (Duroc × Large White/Landrace) were used in this study. Within 16 h of birth (PND 0), pigs from 7 litters were randomly assigned to receive either AB (Amoxicillin: 30 mg/kg/day, *n* = 6/litter) or PL (antibiotic flavoring, *n* = 6/litter) with treatments being divided equally between males and females (3 males and 3 females per sow per treatment). Pigs receiving AB and PL within each litter were co-housed together and suckled from their maternal sows. Pigs received their respective treatment by oral gavage split between two doses at 0800 and 01600 h from post-natal day (PND) 0 to 14. Post-natal day is defined as the age of pig in days post-birth. All animals were maintained on a 12 h photoperiod with room temperature of 22–25°C and allowed *ad libitum* access to the sow for milk and water between PND 0–14. Three piglets were removed from the experiment (2 from AB group and 1 from PL group) due to physical injuries unrelated to experimental treatment.

### Fecal and Cecal Sample Collection

On PND 3, 7, 14, 21, 35, and 49 fecal swabs were taken from live pigs for 16S rRNA characterization of microbial community composition. Fecal swabs were held at −80°C until further processing. On PND 7 (*n* = 6/treatment), PND 14 (AB, *n* = 8; PL, *n* = 6) and PND 21 (*n* = 5/treatment) pigs were euthanized by captive bolt and cecal digesta was collected and dilutions were plated on selective media (MacConkey agar) and incubated for 18 h at 37°C to enumerate live cecal coliforms. Cecal digesta was only used for enumeration of coliforms, whereas fecal swabs were used for 16S rRNA gene amplicon sequencing.

### Immune Cell Isolation and Phenotype Analysis

On PND 21 and 49, whole blood was collected to extract PBMCs for assessing immune cell phenotype. PBMCs were purified from blood collected in EDTA tubes using Histopaque 1077 (Sigma-Aldrich, St. Louis, MO) density centrifugation (25). Cells were isolated by passing tissue through a 100 nm nylon mesh under sterile conditions to obtain a single cell suspension (26). Erythrocytes were lysed with ammonium chloride lysis buffer (155 mM NH_4_Cl, 0.1 mM EDTA, 10 mM KHCO_3_; Fisher Scientific, Edmonton, AB, Canada). Cells were washed and re-suspended in complete RPMI 1640 medium supplemented with 5% heat-inactivated fetal calf serum, 25 mM HEPES, 2.5 mM 2-mercaptoethanol and 1% antibiotic/antimycotic (pH 7.4) (Invitrogen, Burlington, ON, Canada). Cells were adjusted to a concentration of 1 × 10^6^ cells/mL.

Immune cell subsets were identified by direct immunofluorescence assay, as previously described (26) and consistent with manufacturer's instructions. Briefly, after a 30 min incubation at 4°C with pre-labeled monoclonal antibodies (Supplementary Table 1), cells were washed then fixed in PBS with 1% w/v paraformaldehyde. The only exception was for Foxp3 where the cells were treated to permeabilize the membrane prior to adding the antibody, as described by the manufacturer. All fixed cells were analyzed within 72 h by flow cytometry (FACSCanto; Becton Dickinson, San Jose, CA, USA) according to the relative fluorescence intensity using Kaluza Software (Beckman Coulter, Mississauga, ON, Canada).

### *Ex vivo* Cytokine Secretion by Mitogen-Stimulated Lymphocytes

Cytokine production by PBMCs was measured as previously described (26). Briefly, PBMCs (1 × 10^6^ cells/mL) were cultured at 37°C for 48 h without mitogen (unstimulated cells) or with phytohaemagglutinin (PHA, 25 μg/mL for PBMC's; Sigma), as it takes 48 h to induce peak cytokine response. Cells were centrifuged for 10 min at 1,500 rpm and the supernatants kept at −80°C. Commercial ELISA kits were used to measure IL-2, IL-10, and IFNγ concentrations according to the manufacturer's instructions (R&D systems, Minneapolis, MN, USA). All detection limits were 15.63–4,000 pg/mL, except for IFNγ, which was 9.76–2,500 pg/mL.

### Intraperitoneal *Salmonella* Typhimurium Challenge

*Salmonella enterica* serovar Typhimurium strain X4232 was cultured on XLD (xylose lysine deoxycholate agar) at 37°C for 24 h. For culture in broth, one isolated colony from *Salmonella enterica* serovar Typhimurium strain X4232 grown on XLD plates was inoculated into LB broth and incubated at 37°C at 150 rpm for 22 h. A 1 ml sample of culture was used to determine CFU/ml by plating a 10^−3^ to 10^−9^ dilution series on XLD agar. The remaining culture was heat-inactivated at 80°C for 1 h.

A sub-group of 41 pigs that were treated with AB (*n* = 20) and PL (*n* = 21), described above, were used for the intraperitoneal *Salmonella* challenge. On PND 49, whole blood was collected from pigs using PAXgene® blood RNA tubes (Qiagen) prior to IP *Salmonella* challenge (0 h). Pigs were randomized into 3 groups (*n* = 6–7/treatment/time) to assess immune response kinetics at 0, 4, and 12 h post IP *Salmonella* challenge. Pigs allocated to 0 h received a saline injection whereas 4 and 12 h groups were injected with 1 ml of heat-inactivated *Salmonella enterica* serovar Typhimurium strain X4232 (1 × 10^9^ CFU/ml) into their IP cavity (lower left abdominal quadrant, 5 cm cephalic to pubis, 5 cm lateral of midline). At 4 h (*n* = 7/treatment) and 12 h (*n* = 7/treatment) post challenge, blood was sampled using PAXgene® blood RNA tubes and pigs were euthanized via captive bolt. To collect infiltrating immune cells an IP lavage was performed by inserting 500 mL of ice-cold sterile PBS^−/−^ (no calcium, no magnesium) into the cavity via a 5” incision into the lower left abdominal quadrant. After 1 min of washing the IP cavity, lavage fluid with infiltrating immune cells was collected and kept on ice for further analysis.

### Peritoneal Infiltrated Leukocyte Counts

Leukocytes infiltrated to the peritoneal cavity were assessed using the hemacytometer method. Briefly, after homogenization, 10 μl of peritoneal lavage were placed in hemacytometer and counted under inverted light microscopy. Values obtained were multiplied by 10,000 and the total volume of the obtained peritoneal lavage. Final numbers represent the total leukocyte infiltration at each time point post *Salmonella* challenge.

### ROS Production

ROS production was estimated by ROS-producing cell number, which was determined based on the CellROX oxidation procedure according to the manufacture's specifications. Briefly, 1 x 10^6^ intra-abdominal leukocytes were incubated with 5 uM of CellROX reagent (Molecular Probes, ThermoFisher Scientific), following by 30 min incubation at 37°C. Following incubation, leukocytes were washed twice with PBS and fixed in 1% formaldehyde. Hoechst 33342 (Molecular Probes) was used for nuclear staining. Ten thousand events were recorded by Imaging Flow cytometry. The numbers of cell positive for CellROX was counted.

To further assess ROS production capacity of intra-abdominal leukocytes from non-challenged animals (0 h) and challenged animals (4 and 12 h), heat-killed *Salmonella enterica* Typhimurium X4232 was added to leukocytes and incubated *ex vivo* for 30 min at 37°C, then ROS production was measured as described above.

### NF-κB Translocation

Peritoneal leukocytes were harvested by lavage at 0 (saline alone), 4, and 12 h post IP *Salmonella* challenge. Leukocytes were fixed in 1% formaldehyde. Fixed cells were washed twice in permeabilization buffer (PBS, 2% FCS and saponin 0.1%) followed by incubation with unlabeled rabbit IgG anti-mouse NF-κB p65 (Santa Cruz Biotechnologies, Dallas, TX) for 30 min on ice and 20 min at room temperature. FITC goat anti-rabbit IgG (Jackson Immunoresearch Laboratories, Inc., Baltimore, MD) were added and incubated for 20 min. Draq5 was used for nuclear staining. Imaging Flow Cytometry was used to record 10,000 events (ImageStream®^X^ Mk II, MilliporeSigma). Ideas software was used to measure co-localization values of NF-κB and Draq5. Events with high NF-κB and Draq5 co-localization values indicate high NF-κB translocation. In contrast, events with low co-localization values indicate low NF-κB translocation.

### RNA Isolation and qPCR

RNA from whole blood was extracted with PAXgene® blood RNA kits (Qiagen®, Valencia, CA) as per instructions. Quantity of RNA was determined using a Nanodrop 2000 (ThermoScientific™, Waltham MA). Maxima® first strand cDNA synthesis kit (ThermoScientific™) was used to synthesize cDNA with 2,000 ng of starting RNA. A Beckman BioMek 3000 liquid handling robot was used to set up qPCR reactions into 384 well plates using specific gene primers (27) (Supplementary Table 2) and 2X perfeCTA™ SYBR® Green SuperMix, ROX™ (Quantabio, Beverly, MA). qPCR reactions were run using an ABI ViiA7 with a holding stage of 3 min at 95°C, followed by 40 cycles of 10 s at 95°C, 30 s at annealing temperature and 30 s at 72°C. Gene expression was calculated using ΔΔCt method with TBP (TATA box binding protein) used as a house keeping gene (28).

### DNA Isolation and 16S Sequencing

Genomic DNA was extracted from fecal swabs using QIAamp® FAST DNA stool mini kit according to manufacturer's instructions (Qiagen®, Valencia, CA). A 1 min bead-beating step was added to facilitate the lysis of gram-positive bacteria. DNA concentrations were measured by Nano-Drop spectrophotometer system ND-1000 (ThermoScientific™, Waltham, MA), purity was assessed by determining the ratio of absorbance at 260 and 280 nm. Sequence library preparation was completed according to Illumina® Miseq protocol.

Sequence data was analyzed using a QIIME pipeline (MacQIIME 1.8.0 OS10.10) (29). PANDAseq was used for quality filtering and to assemble the paired end reads into contigs with miscalled or uncalled bases discarded (30). Resulting sequences were cleared of chimeras and singletons using UCHIME and UPARSE workflows, respectively, and were clustered into operational taxonomic units (OTUs) having >97% similarity with USEARCH (31–33). Taxonomy was assigned using Ribosomal Database Project (RDP) classifier V2 (34). Alpha diversity and beta diversity estimations were conducted using the QIIME workflow core_diversity_analysis.py (35).

### Statistical Analysis

All data was tested for normality using Kolmogorov-Smirnov test. When non-normal distribution occurred, non-parametric Kruskall-Wallis test was used with *post-hoc* pair-wise comparison made using the Dwass, Steel, Cirtchlow-Fliger multiple comparison procedure (SAS, University Edition). When normal distribution occurred one-way, and two-way ANOVAs were performed with *post-hoc* pairwise comparisons made with a Bonferroni correction using GraphPad Prism v6.02 (La Jolla, CA). Principle coordinate analysis of genus level sequence data was performed on R (v3.2.1) using the Bray Curtis dissimilarity. Statistical significance was expressed as *P* < 0.05 with a *P*-value between 0.1 and 0.05 considered a trend.

## Results

### Peripheral Blood Immune Cell Phenotype and Function Altered With Early Life Antibiotic Treatment of Pigs

**Figure 1:**
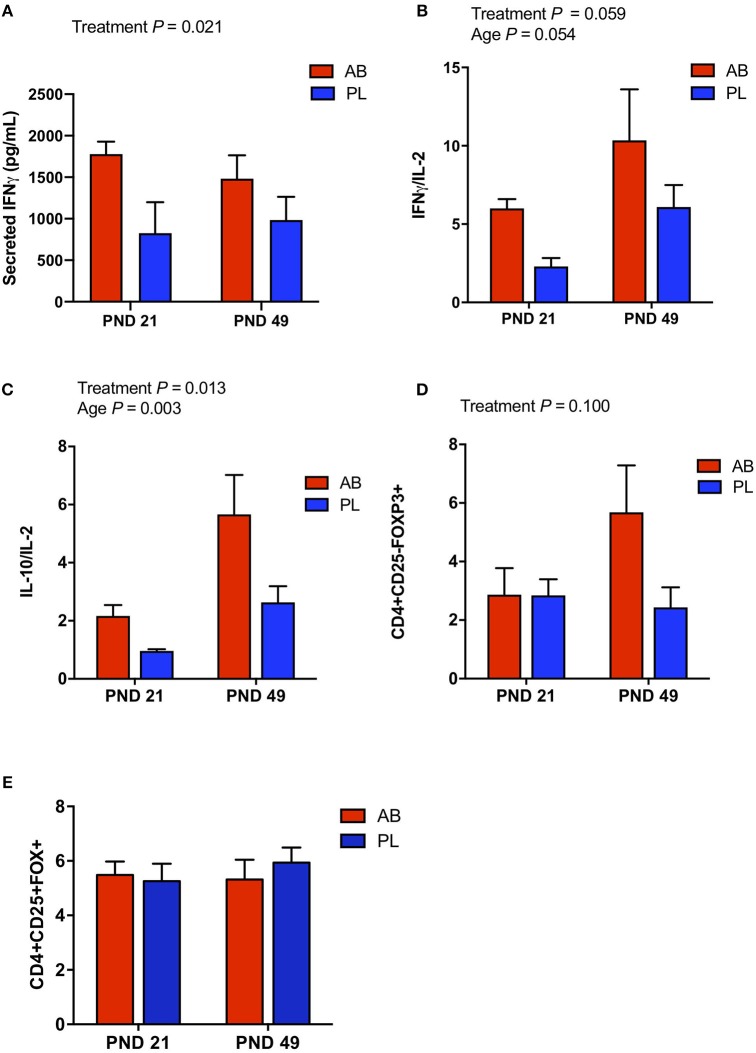
*In vitro* cytokine secretion and cell sub-populations of PBMC were affected by antibiotic (AB) treatment. PBMCs from AB and placebo (PL) treated pigs were stimulated *in vitro* with phytohaemagglutinin (PHA), a T cell mitogen. **(A)** Secreted IFNγ was higher in AB vs. PL treated pigs (Two-way ANOVA, Treatment *P* = 0.006, Age *P* = 0.022, Interaction *P* = 0.472). **(B)** The ratio of IFNγ/IL-2 tended to be higher in AB vs. PL treated pigs and tended to increase with age (Two-way ANOVA, Treatment *P* = 0.059, Age *P* = 0.054, Interaction *P* = 0.780). **(C)** The ratio of IL-10/IL-2 was higher in AB vs. PL treated pigs and increased with age (Two-way ANOVA, Treatment *P* = 0.013, Age *P* = 0.003, Interaction *P* = 0.248). **(D)** In addition, immune cell subpopulation CD4+CD25-FoxP3+, identified by flow cytometry, tended to be higher in AB treated pigs (Two-way ANOVA, Treatment *P* = 0.100, Age *P* = 0.220, Interaction *P* = 0.106. **(E)** However, the CD4+CD25+FoxP3+ immune cell subpopulation was not affected by treatment or age (Two-way ANOVA, Treatment *P* = 0.752, Age *P* = 0.684, Interaction *P* = 0.500). PND 21: AB *n* = 7, PL *n* = 7; PND 49: AB *n* = 7, PL *n* = 8; PND 84: AB *n* = 8, PL *n* = 7.

Peripheral blood mononuclear cells (PBMCs) were isolated on PND 21 and 49 to assess how neonatal AB treatment affects immune cell population and function *in vivo*. Overall, the percentage of CD3^+^CD4^+^ T cells among the broader T cell population (CD3^+^CD4^+^/CD3^+^) was significantly higher (Table 1; *P* < 0.05) in AB pigs compared to PL pigs at PND 21. In addition, on PND 21 AB treated pigs had a reduced number of CD4^+^CD45RA^+^ (naïve) T cells per liter of blood (Table 1; *P* < 0.05), and tended to have an increased percentage of CD4^+^CD45RA^−^ T cells (Table 1; *P* < 0.1), which were likely to be CD4^+^CD45RO^+^ (memory) T cells, suggesting a higher concentration of effector/memory T cells. The percentage of γδ T cells in CD3+ T cells tended to be increased in AB pigs compared to PL pigs (Table 1; *P* = 0.08). When stimulated *in vitro* with phytohaemagglutinin (PHA), a polyclonal T cell mitogen, PBMCs from both treatments at PND 21 and 49 produced similar levels of IL-2 (Supplementary Table 3), whereas AB treated pigs secreted significantly higher levels of IFNγ (Figure 1A; *P* < 0.05) compared to PL. IL-10 and IL-6 production did not differ between treatments on PND 21 and 49 (Supplementary Table 3). Notably, the ratio of IL-10 to IL-2, reflecting the immune-regulatory/-suppressive signaling, was higher with AB treatment (Figure 1C; *P* = 0.013). Additionally, CD4^+^CD25^−^FoxP3^+^ cells tended to be higher in AB pigs at PND 49 (Figure 1D; *P* = 0.100); however, CD4^+^CD25^+^FoxP3^+^ cells did not differ between treatments (Figure 1E; *P* = 0.752).

**Table 1:** Peripheral blood immune cell populations of AB and PL pigs on PND 21 and 49^1^.

	**PND 21**		**PND 49**		**SEM**	***P-*****values**		
	**PL**	**AB**	**PL**	**AB**		**(Treatment)**	**(Age)**	**(Treatment × Age)**
**Cell percentage (% OF 10**^**6**^ **cells)**								
CD3^+^	52.6	48.3	50.1	46.8	1.68	0.285	0.570	0.892
CD3^+^CD4^+^	17.1	20.5^*^	15.1	14.9	0.838	0.226	0.007	0.184
CD3^+^CD8^+^	11.8	11.5	8.7	8.7	0.731	0.857	0.018	0.893
CD4^+^CD45RA^+^	12.6	9.1^a^	3.5	5.5	0.731	0.618	<0.001	0.078
CD4^+^CD45RA^−^	12.5	18.0^a^	18.4	15.4	2.20	0.582	0.456	0.066
γδ	12.7	15.1	22.1	22.5	1.80	0.446	<0.001	0.573
**Cell counts (×10**^**9**^**/L blood)**								
CD3^+^	2.10	1.40	3.62	3.25	0.236	0.143	<0.001	0.636
CD3^+^CD4^+^	0.68	0.59	1.10	1.02	0.049	0.503	0.002	0.985
CD3^+^CD8^+^	0.47	0.32	0.67	0.60	0.064	0.331	0.048	0.735
CD4^+^CD45RA^+^	0.51	0.27^*^	0.25	0.37	0.043	0.441	0.331	0.036
CD4^+^CD45RA^−^	0.47	0.52	1.33	1.08	0.150	0.478	<0.001	0.308
γδ	0.50	0.44	1.56	1.57	0.150	0.840	<0.001	0.814
**Cell subtype ratio (%)**								
CD4^+^/CD8^+^	132.8	142.7	154.9	154.3	4.68	0.525	0.027	0.476
CD3^+^CD4^+^/CD3^+^	32.3	39.8^*^	30.1	31.8	1.72	0.031	0.017	0.163
CD3^+^CD8^+^/CD3^+^	22.0	23.8	17.3	18.4^*^	1.25	0.449	0.012	0.844
CD4^+^CD45RA^+^/CD4^+^	51.8	33.6^a^	17.4	26.6	3.97	0.515	0.006	0.056
γδ /CD3^+^	24.0	30.2	44.1	47.8	2.90	0.087^a^	<0.001	0.655

1 Data presented as mean ± pooled SEM.

* P < 0.05 and

a *P ≤ 0.10 comparing AB to PL within the same age group. Data was analyzed using a Two-way ANOVA with post-hoc comparisons between treatments within the same age group corrected using Bonferroni. AB, antibiotic; PL, placebo; PND, post-natal day; SEM, pooled standard error of the mean. PND 21 n = 7/treatment, PND 49 n = 7/treatment*.

### Treatment With Antibiotics Resulted in Transient Changes in Fecal Microbial Composition

**Figure 2:**
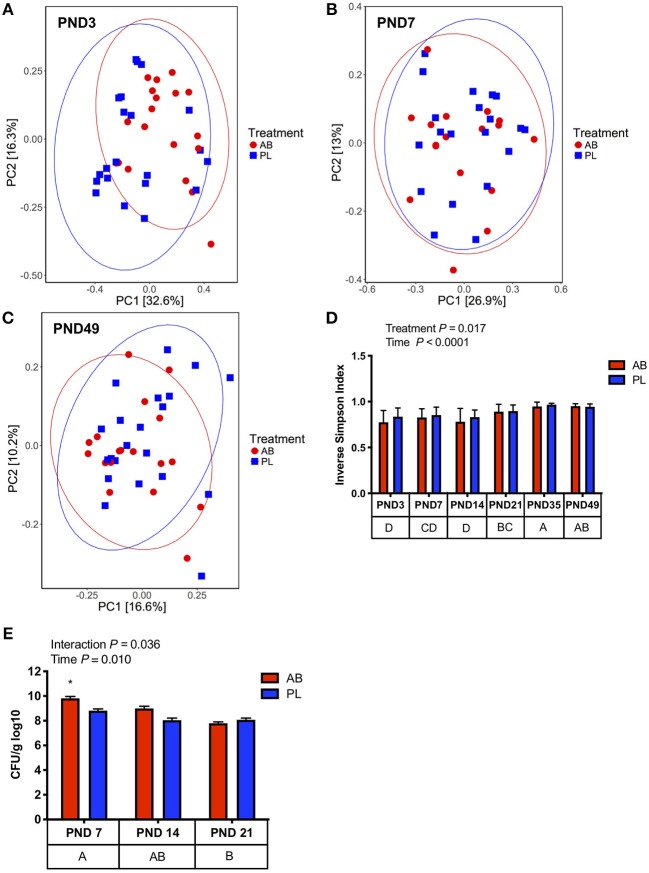
Pigs treated with antibiotics (AB) from day 0–14 had transient changes in microbial community composition compared to those treated with placebo (PL), as determined with Bray-Curtis dissimilarity. **(A)** On PND 3 there is distinct clustering of pigs treated with AB vs. PL (Adonis, *P* = 0.025); AB *n* = 20, PL *n* = 21; **(B)** On PND 7 no distinct clustering was observed between pigs treated with AB and PL (Adonis, *P* = 0.693); AB *n* = 17, PL *n* = 20. **(C)** On PND 49 pigs treated with AB and PL were not distinctly clustered (Adonis, *P* = 1.0) **(D)** Alpha diversity was reduced in AB treated pigs and significantly increased overtime (Two-way ANOVA, Treatment *P* = 0.017; Time *P* < 0.0001; Interaction *P* = 0.444). PND 14: AB *n* = 18, PL *n* = 21; PND 21: AB *n* = 18, PL *n* = 21; PND 35: AB *n* = 20, PL *n* = 21; PND 49: AB *n* = 18, PL *n* = 21. **(E)** There was a significant interaction between Treatment and Day on cecal coliform counts. Pigs treated with AB on PND 7 had higher coliform counts vs. PL on PND 7, 14, and 21 and AB on PND 14 and 21 as indicated by an asterisk (Two-way ANOVA; Treatment *P* = 0.0257; Day *P* = 0.0103; Interaction *P* = 0.0358, *post-hoc* pair-wise comparisons made with Bonferroni correction), PND 7: AB *n* = 6, PL *n* = 6; PND 14: AB *n* = 8, PL *n* = 6; PND 21: AB *n* = 5, PL *n* = 5. The main effect of time is displayed below each figure and those PND not sharing a common letter differ significantly.

Fecal microbial community structure was altered with AB on PND 3 as shown by a distinct clustering between AB vs. PL treated pigs (Figure 2A, Adonis, *P* = 0.025). However, AB only transiently altered microbial composition, whereby on PND 7 to 49 AB and PL treated pigs no longer had distinct microbial structures (Figures 2B,C; Supplementary Figures 1A–C). Distinct clustering of pigs treated with AB on PND 3 was in part due to a 48% reduction of *Firmicutes* and 66% increase in *Proteobacteria* (Supplementary Table 4). Higher *Proteobacteria* in AB treated pigs was mainly due to a bloom of *Enterobacteriaceae* (29.3 vs. 14.3%, Supplementary Table 4). Overall, AB treatment reduced α-diversity (Figure 2D, Inverse Simpson Index, *P* = 0.017). After PND 3 normal successional patterns were observed between pigs treated with AB and PL with age being the main driver of microbial community composition (Adonis, *P* < 0.01; Supplementary Figure 2A). The predominant phyla changing with age was the increasing relative abundance of *Firmicutes* at the expense of *Proteobacteria* (Supplementary Table 5; Supplementary Figure 2C). Characteristic successional patterns were also observed for α-diversity, which increased from PND 3 to PND 35 as determined by Shannon diversity index (Supplementary Figure 2B). To confirm the higher *Enterobacteriaceae*, total coliforms were enumerated in cecal samples collected on PND 7, 14, and 21. Mean total cecal coliform counts were 9.81 vs. 8.82 log_10_ CFU/g in AB vs. PL pigs on PND 7, and similar to sequence data normalized after AB withdrawal (Figure 2E).

### Early Life Antibiotic Exposure Upregulates Systemic Pro-inflammatory Gene Expression Following *in vivo Salmonella* Challenge

**Figure 3:**
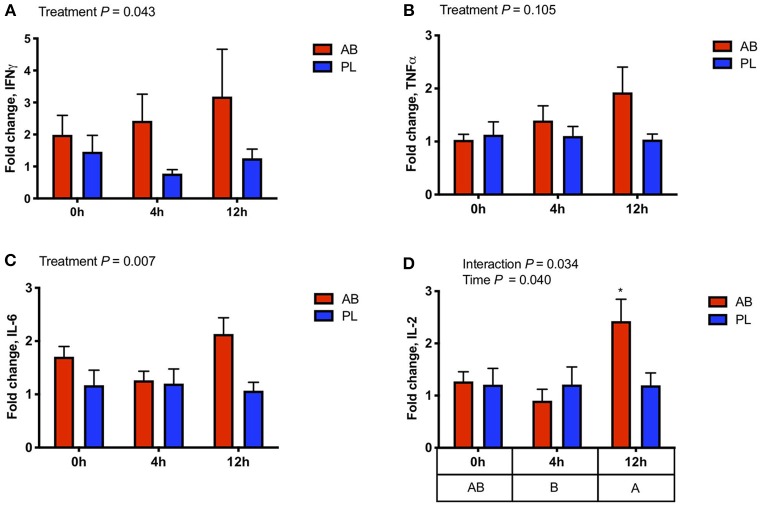
Gene expression of effector cytokines in whole blood was altered 0, 4, and 12 h post intraperitoneal *Salmonella* challenge in pigs treated with antibiotics (AB) vs. placebo (PL) as analyzed by a Two-way ANOVA **(A)** IFNγ expression was higher in AB vs. PL treated pigs (Treatment *P* = 0.043, Time *P* = 0.715, Interaction *P* = 0.647). **(B)** TNFα expression tended to be higher with AB treatment (Treatment *P* = 0.105, Time *P* = 0.192, Interaction *P* = 0.338). **(C)** IL-6 expression was also higher in AB vs. PL treated pigs (Treatment *P* = 0.007, Time *P* = 0.312, Interaction *P* = 0.125). **(D)** There was an interaction between time and treatment on the expression of IL-2 and was increased in AB treated pigs 12 h post-challenge compared to AB and PL treated pigs at all time points as indicated by an asterisk (Treatment *P* = 0.186; Time *P* = 0.040, Interaction *P* = 0.034) *n* = 7/treatment. The main effect of time is displayed below the figure and those time points not sharing a common letter differ significantly. *Post-hoc* pair-wise comparisons made with Bonferroni correction.

Peripheral blood gene expression of IFNγ, TNFα, IL-6, and IL-2 were measured at 0, 4, and 12 h post *S*. Typhimurium challenge to assess if AB treatment had an effect on classical pro-inflammatory cytokines. Gene expression of IFNγ, a prominent Th1 effector cytokine, was upregulated (Figure 3A; *P* = 0.043) in AB treated pigs when compared with pigs treated with PL. Furthermore, IL-6 and IL-2, key players in inflammatory responses, had higher expression in pigs exposed to AB vs. PL (Figure 3C; *P* = 0.007; Figure 3D; *P* = 0.034, respectively). TNFα gene expression also tended to be higher in AB compared to PL treated pigs (Figure 3B; *P* = 0.10). These results reveal that AB exposure induces upregulation of pro-inflammatory mediators in peripheral leukocytes during early immune responses against *S*. Typhimurium challenge.

### Early Life Antibiotic Exposure Promotes a Stronger Local Immune Response Following *in vivo Salmonella* Challenge

**Figure 4:**
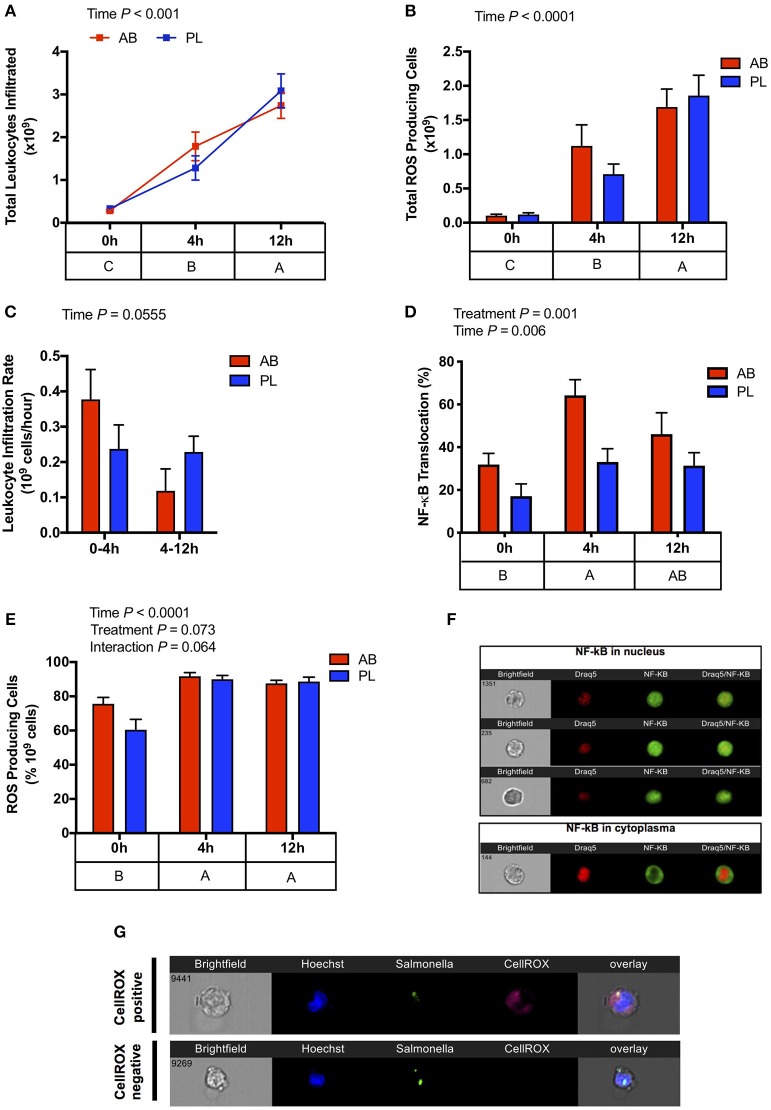
Antibiotic (AB) exposure induced a stronger leukocyte activation against an intraperitoneal *Salmonella* challenge in comparison to pigs treated with placebo (PL). **(A)** Total leukocyte infiltration (Two-way ANOVA Treatment *P* = 0.870, Time *P* < 0.001, Interaction *P* = 0.306) and **(B)** and total reactive oxygen species producing cells (Two-way ANOVA Treatment *P* = 0.963, Time *P* = 0.009, Interaction *P* = 0.845) increased progressively 4 and 12 h post IP *Salmonella* challenge. **(C)** Leukocyte infiltration tended to reduce over time (Two-way ANOVA Treatment *P* = 0.823, Time *P* = 0.055, Interaction *P* = 0.072). **(D)** Leukocyte activation detected through NF-κB nuclear translocation was higher in the AB treated pigs compared to PL post *Salmonella* challenge, and increased over time (Two-way ANOVA Treatment *P* = 0.001, Time *P* = 0.006, Interaction *P* = 0.410). **(E)** Exposure of peritoneal leukocytes *in vitro* to heat-killed *Salmonella* resulted in increased ROS production in both the AB and PL treated pigs between 0 and 4 h and 0 and 12 h with a tendency of AB treated piglets to have higher ROS production (Two-way ANOVA Treatment *P* = 0.073, Time *P* < 0.01, Interaction *P* = 0.064). *n* = 4–5/treatment/time. **(F)** Representative images of neutrophil NF-κB nuclear translocations **(G)** Representative images of ROS production in response to *in vitro* stimulation. The main effect of time is displayed below each figure and those PND not sharing a common subscript differ significantly.

To directly measure whether AB exposure affected acute immune response to a pathogen, heat killed *S*. Typhimurium was injected (IP) into the abdominal cavity of AB and PL pigs, and leukocyte infiltration and reactive oxygen species (ROS) production were measured at time 0, 4, and 12 h after challenge. Although number of infiltrating leukocytes and ROS production increased over time, values did not differ between AB and PL groups (Figures 4A,B). Leukocyte infiltration rate tended to reduce during 4–12 h compared to 0–4 h post-challenge in both AB and PL pigs (Figure 4C; *P* = 0.055). Interestingly, leukocytes extracted from AB pigs had upregulated NF-κB nuclear translocation compared to PL pigs (Figures 4D,F; *P* = 0.001). On the other hand, *in vitro* phagocytic index did not differ between treatments (data not shown). When extracted leukocytes from non-challenged and challenged pigs were further stimulated *in vitro* with heat-killed *S*. Typhimurium ROS production was elevated from both AB and PL groups over time (*P* < 0.0001) and tended to be increased in AB vs. PL animals (*P* = 0.073) (Figures 4E,G).

## Discussion

It is well-established that microbial colonization during the neonatal period plays a major role on immune development and function, with perturbations in colonization during childhood commonly associated with immune mediated disorders later in life. However, to date, limited research has assessed how these antibiotic induced microbial changes alter host immune development and response to an immune challenge. In the current study, we observed that early life amoxicillin exposure in pigs altered PBMC phenotype after antibiotics had been withdrawn. Analyses of PBMC subsets showed that pigs treated with AB had a higher proportion of CD3^+^CD4^+^ T cells in total T cells compared with PL, whereas the proportion of CD4^+^CD45RA^+^ (naïve) cells was less in AB vs. PL pigs on PND 21. In the meantime, the proportion of CD4^+^CD45RA^+^ cells in total PBMC and total CD4^+^ T cells tended to be lower in AB vs. PL treated pigs on PND 21. Thus, it is possible that the increase in CD3+CD4+ T cells could be caused by a higher proportion of CD4^+^CD45RO^+^ (memory) cells at PND 21. This was not directly tested, however, we observed a trend toward increased percentage of CD4^+^CD45RA^−^ T cells (*P* < 0.1), which were likely to be CD4^+^CD45RO^+^ (memory) T cells. It is possible that AB treatment killed a large number of commensal bacteria, releasing antigens for immune recognition, which amplified inflammatory responses and the development of immune memory that could promote pro-inflammatory rather than immunoregulatory responses (36). It is also reported that early-life antibiotics can affect the function and increase the pathogenicity of CD4^+^ T cells (37). Indeed, significantly higher amounts of IFNγ were produced by PBMCs from AB pigs when stimulated with a T-cell mitogen *in vitro* at PND 21 and 49. At the same time, the ratio of IL-10 to IL-2 was higher in AB pigs compared to PL pigs, likely due to a marginal increase in IL-10, as IL-2 production did not differ. It has been reported that human γδ T cells secrete little IL-2 but high concentrations of IFN-γ and IL-10 (38). In the current study, the percentage of γδ T cells in CD3+ T cells had a tendency to be higher in AB pigs compared to PL pigs (*P* = 0.087), potentially contributing to IFNγ production in the absence of more IL-2 (Figure 1B). This is an advantage of the pig model, as pigs have similar γδ T cell tissue distribution as humans but a higher percentage in circulation (39). In contrast, mice have certain oligoclonal γδ T cells exclusive to tissues despite the similar percentage of the circulating T cell pool vs. human (39). In the current study, the proportion of CD4^+^CD25^+^FoxP3^+^ cells between treatments did not differ. However, at PND 49, CD4^+^CD25^−^FoxP3^+^ cells, a group of T regulatory cells developed from naïve CD4^+^ T cells that can secrete IL-10 without IL-2 induction, tended to be higher in AB pigs. This cell subset could also contribute to the higher IL-10/IL-2 ratio observed when PBMCs stimulated with mitogen *in vitro*. It is worth to note that, with marginally increased IL-10 production and a higher IL-10/IL-2 ratio, PBMCs from AB pigs were still able to secrete a significantly higher amount of IFNγ. We speculate that AB treatment might promote the development of pro-inflammatory immune system, in which immunoregulatory responses were not strong enough to dampen inflammatory signaling (40).

To investigate long-term implications of early antibiotic exposure on immune function, an IP *Salmonella* challenge was used to observe immune kinetics of animals at 0, 4, and 12 h post-challenge on PND 49. As expected, challenged pigs showed marked recruitment of leukocytes into the peritoneal cavity as early as 4 h with highest levels of infiltration occurring at 12 h post-challenge. This was paralleled by enhancements in the number of leukocytes with a capacity to produce ROS. However, in both cases, no differences were detected between AB and PL groups. However, increased IFNγ gene expression in blood of AB treated animals was observed post-challenge, along with increased pro-inflammatory mediators IL-6 and TNFα, indicating a more prominent Th1 response. The NF-κB signaling pathway is critical for T cell IFNγ production (41). Interestingly, assessment of NF-κB nuclear translocation in activated peritoneal leukocytes of AB treated pigs showed a greater and earlier capacity of activation post-challenge and coincided with the upregulated PBMC IFNγ expression. The increased IL-2 expression in AB pigs 12 h post-challenge may suggest positive implications for systemic long-term immunity, as IL-2 can promote memory T cell development (42). *In vitro* stimulation of leukocytes from the IP cavity of unchallenged (0 h) and challenged (4 and 12 h) pigs with *Salmonella* further demonstrated that AB treated pigs had more rapid activation of leukocytes. Taken together, this indicates AB treated animals displayed higher leukocyte activation and further Th1 type immune response. These results suggest that early AB exposure induces changes in local and systemic immune response regulation, increasing expression of pro-inflammatory mediators and modulating leukocyte functionality. Antibiotic treatment can result in elevation of inflammatory markers in the intestine of mice (43, 44) priming the immune system toward an inflammatory state, which may explain the more rapid response in AB treated pigs vs. PL post IP *Salmonella* challenge. The changes observed in immune response induced by AB is potentially mediated through alterations in resident gut microbiota. Research has shown distinct microbiota members can prevent or reduce severity of *Salmonella* infection (45, 46). Priming of the mucosal immune system through enhanced bacterial induced CD3^−^ and CD4^+^ T IFNγ responses was found to be one mechanism altering disease resistance (47). Recently, IFNγ and TNFα cytokine production capacity has been linked to differential abundance of specific gut bacteria, suggesting that microbial composition plays a key role in cytokine production and subsequent disease resistance (48).

In the current study, increased *Enterobacteriaceae* of AB treated pigs, was detectable up to PND 7 (Figure 2E). Established in the 1950's to 60's, antibiotic treatment was found to expand colonization of *Enterobacteriaceae* (49, 50). In agreement, exposure of human infants to broad-spectrum antibiotics within 48 h of birth increases *Enterobacteriaceae* up to 2 months post-administration (51, 52). However, diversity and successional patterns returned to normal after PND 3 in the current study, as determined with an inverse Simpson index (Figure 3D) and indistinct clustering of AB and PL treated animals (PCoA, *P* > 0.05; Figures 3B,C, Supplementary Figures 2A–C). The quick return of pigs treated with AB to indistinguishable successional patterns to their PL treated littermates may be a function of co-housing of the treatment groups. However, AB still triggered long-lasting systemic and local immune functional changes. This implies that there may be a small window of time during neonatal development where immune education occurs through gut-associated microbiota that may not be reversible through reconstitution of the microbiota profile. Future studies will be required to determine whether the altered immune phenotypes are an indirect result of enriched *Enterobacteriaceae*, other microbiome alterations, or a direct effect of amoxicillin.

The results presented in this study reflect the responses in young and developing animals. We have observed that PBMCs isolated from AB pigs show increased IFNγ production in response to PHA stimulation up to PND 84 (data not shown). We have not yet looked beyond PND 84, therefore it remains unclear whether these effects on immune function would be observed past early development into adulthood. Epidemiological data following children from birth has shown that antibiotic exposure within the first year of life is associated with increased risk of asthma development with diagnoses detected up to 9 years of age (53). Another population-based cohort study demonstrated that early childhood antibiotic exposure was associated with development of IBD (54). These studies suggest there may be long-term effects of neonatal antibiotics that extend past early childhood. This is of critical importance to the treatment of young children as amoxicillin is one of the most heavily prescribed antibiotics.

Overall, this study shows despite the transient nature of the antibiotic effect on the microbiome, early life amoxicillin exposure can alter immune response locally and systemically, long after AB withdrawal. Those piglets treated with antibiotics showed more rapid and pronounced pro-inflammatory responses to the IP challenge compared to piglets treated with placebo, which might prove beneficial during pathogen infection. However, it is possible that this pro-inflammatory response could result in excessive inflammation, local tissue damage, and possibly increase the risk of immune mediated diseases. Further studies will be required to determine how long this window of immune programming remains open.

## Data Availability

Sequence reads of the 16S rRNA gene amplicon data is available through the National Centre for Biotechnology Information Sequence Read Archive (Accession SRP158254).

## Author Contributions

JF, KY, and JM-B performed the animal study, analyzed the data, and wrote the manuscript. YG helped to perform the animal study. SG was responsible for immune cell characterization and data analysis. CF designed the experimental approach, analyzed the immune data, and reviewed the manuscript for final content. GP contributed to study design, interpretation of results, and reviewed the manuscript for final content. DB and BW designed the experimental approach and reviewed the manuscript for final content.

### Conflict of Interest Statement

The authors declare that the research was conducted in the absence of any commercial or financial relationships that could be construed as a potential conflict of interest.

## Supplementary Material

Supplementary Figure 1 Microbial community structure between pigs treated with antibiotic (AB) vs. placebo (PL) did not differ as determined with a Bray Curtis dissimilarity on **(A)** PND 14 (Adonis, *P* = 0.464); **(B)** PND 21 (Adonis, *P* = 1.0); **(C)** PND 35 (Adonis, *P* = 1.0).

Supplementary Figure 2 Successional patterns are the main driving force of microbial structure in pigs treated with antibiotic (AB) and placebo (PL). **(A)** Bray-Curtis dissimilarity (*P* < 0.01; ADONIS). **(B)** Shannon diversity index on days 3, 7, 14, 21, 35, and 49. Days not sharing a common letter are significantly (*P* < 0.05). **(C)** Predominant phyla in feces of pigs treated with AB or PL on PND 3, 7, 14, 21, 35, and 49. PND 3 *n* = 41; PND 7 *n* = 37; PND 14 *n* = 39; PND 21 *n* = 39; PND 35 *n* = 41; PND 49 *n* = 39.

Supplementary Table 1 PBMC immune cell subset staining panels for flow cytometric analysis.

Supplementary Table 2 Primers used for qPCR gene expression of RNA extracted from whole blood.

Supplementary Table 3 *In vitro* cytokine secretion from PHA stimulated PBMCs between AB and PL piglets on PND 21 and 49^1^. ^1^Data present as mean ± pooled SEM. Data was analyzed using a Two-way ANOVA. AB, antibiotic; PL, placebo; PHA, phytohaemagglutinin; PBMC, peripheral blood mononuclear cell; PND, post-natal day; SEM, pooled standard error of the mean. PND 21: AB *n* = 7, PL *n* = 7; PND 49: AB *n* = 7, PL *n* = 8.

Supplementary Table 4 Relative abundance of predominant fecal genera on PND 3 of AB and PL piglets^1^. ^1^Data presented as mean ± pooled SEM. Data was analyzed using a non-parametric Kruskall Wallis test. AB *n* = 20, PL *n* = 21. AB, antibiotic; PL, placebo; PND, post-natal day; SEM, pooled standard error of the mean; n/d, not detected.

Supplementary Table 5 Relative abundance of predominant fecal phyla of AB an PL pigs on PND 3–49^1^. ^1^Data presented as mean ± pooled SEM. Within a row, mean values without a common superscript differ (*P* < 0.05) or tend to differ (*P* ≤ 0.10). Data was analyzed using a non-parametric Kruskall Wallis test, with *post-hoc* pair-wise comparisons made using Dwass, Steel, Cirtchlow-Fliger multiple comparison procedure. PND 3 *n* = 41, PND 7 *n* = 38, PND 14 *n* = 39, PND 21 *n* = 39, PND 35 *n* = 42, PND 49 *n* = 39. AB, antibiotic; PL, placebo; PND, post-natal day; SEM, pooled standard error of the mean; n/d, not detected.
