# Supplementary figures and images for: Neonatal Exposure to Amoxicillin Alters Long-Term Immune Response Despite Transient Effects on Gut-Microbiota in Piglets
Source: Front Immunol. 2019 Sep 4;10:2059. doi: 10.3389/fimmu.2019.02059 (PMC6737505; doi:10.3389/fimmu.2019.02059)

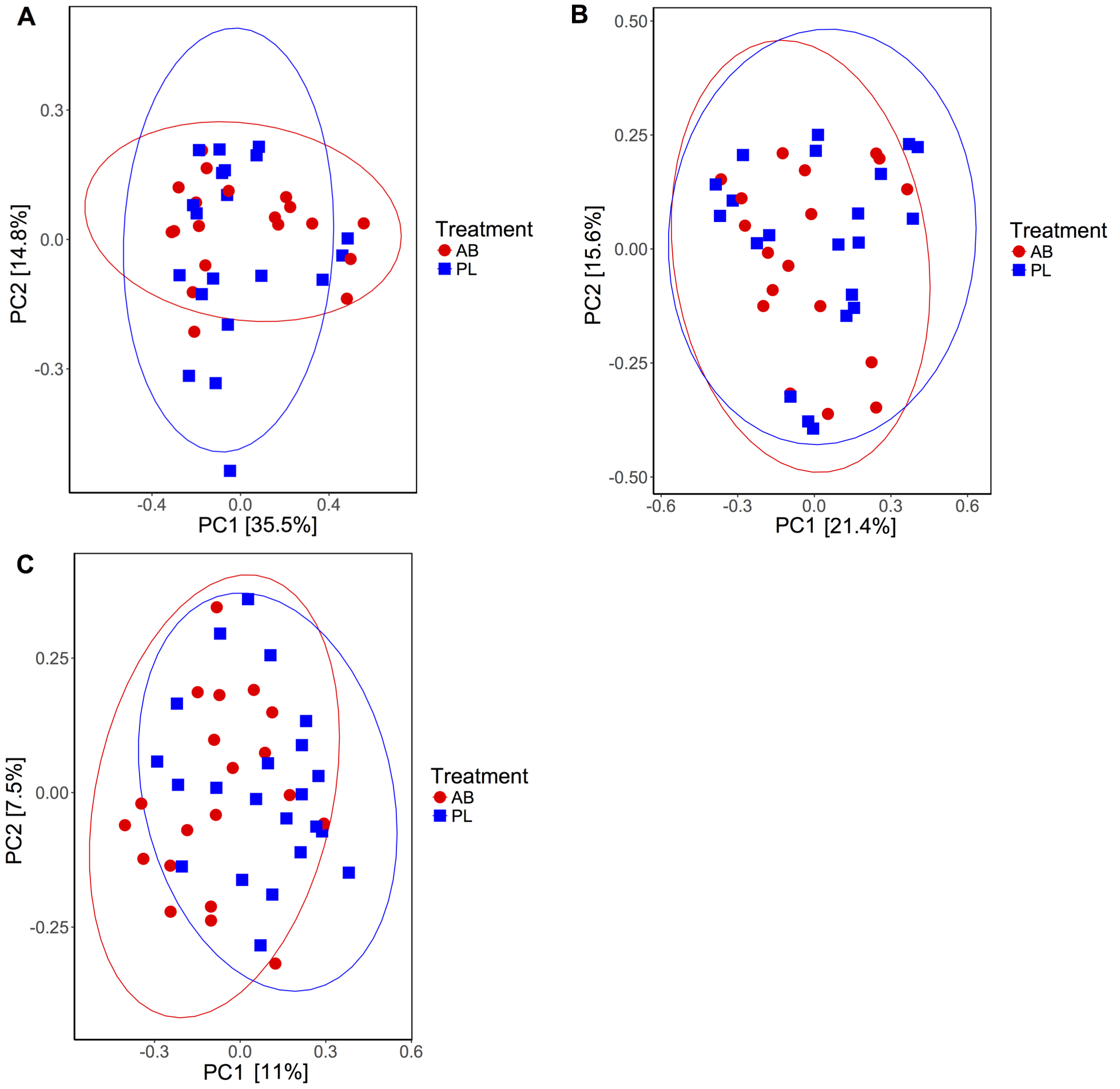

Supplement: Supplementary Figure 1 — Microbial community structure between pigs treated with antibiotic (AB) vs. placebo (PL) did not differ as determined with a Bray Curtis dissimilarity on (A) PND 14 (Adonis, P = 0.464); (B) PND 21 (Adonis, P = 1.0); (C) PND 35 (Adonis, P = 1.0). [file Image_1.TIFF]

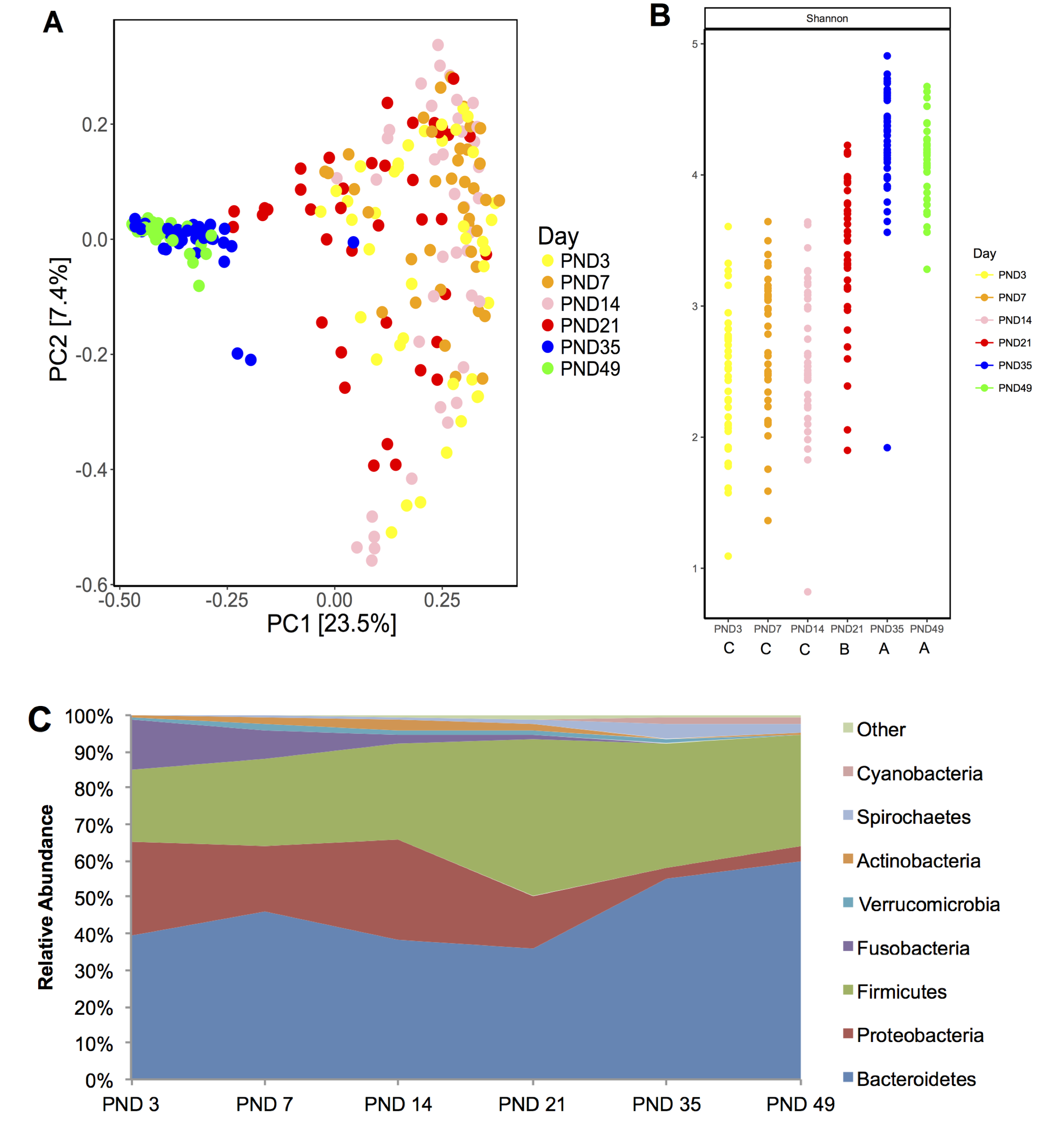

Supplement: Supplementary Figure 2 — Successional patterns are the main driving force of microbial structure in pigs treated with antibiotic (AB) and placebo (PL). (A) Bray-Curtis dissimilarity (P < 0.01; ADONIS). (B) Shannon diversity index on days 3, 7, 14, 21, 35, and 49. Days not sharing a common letter are significantly (P < 0.05). (C) Predominant phyla in feces of pigs treated with AB or PL on PND 3, 7, 14, 21, 35, and 49. PND 3 n = 41; PND 7 n = 37; PND 14 n = 39; PND 21 n = 39; PND 35 n = 41; PND 49 n = 39. [file Image_2.TIFF]
