# Supplementary material for: Neonatal Exposure to Amoxicillin Alters Long-Term Immune Response Despite Transient Effects on Gut-Microbiota in Piglets
Source: Front Immunol. 2019 Sep 4;10:2059. doi: 10.3389/fimmu.2019.02059 (PMC6737505; doi:10.3389/fimmu.2019.02059)
Supplement: Supplementary Table 1 — PBMC immune cell subset staining panels for flow cytometric analysis. [file Table_1.DOCX]

**Supplementary Table 1** PBMC Immune Cell Subset Staining Panels for Flow Cytometric Analysis

| **Panel** | **FITC** | **PE** | **APC** | **PerCP-Cy™5.5** |
| --- | --- | --- | --- | --- |
| **1** | -- | CD3 (BD, 561485) | -- | CD4 (BD, 561474) |
| **2** | CD3 (BD, 559582) | CD8 (BD, 559584) | -- | CD4 (BD, 561474) |
| **3** | -- | CD8 (BD, 559584) | γδ T Lymphocytes (BD 561482) | CD4 (BD, 561474) |
| **4** | CD45RA (Bio-Rad, MCA1751F) | CD8 (BD, 559584) | -- | CD4 (BD, 561474) |
| **5** | CD25 (Bio-Rad, MCA1736) +  Goat anti Mouse IgG, FITC  (Bio-Rad, STAR117F) | -- | FOXP3 (eBioscience™, 17-5773-82) | CD4 (BD, 561474) |
