# Supplementary material for: Neonatal Exposure to Amoxicillin Alters Long-Term Immune Response Despite Transient Effects on Gut-Microbiota in Piglets
Source: Front Immunol. 2019 Sep 4;10:2059. doi: 10.3389/fimmu.2019.02059 (PMC6737505; doi:10.3389/fimmu.2019.02059)
Supplement: Supplementary Table 2 — Primers used for qPCR gene expression of RNA extracted from whole blood. [file Table_2.DOCX]

**Supplemental Table 2** Primers used for qPCR gene expression of RNA extracted from whole blood

| **Gene** | **Forward Primer** | **Reverse Primer** | **Annealing temperature(°C)** | **Reference** |
| --- | --- | --- | --- | --- |
| TBP | AACAGTTCAGTAGTTATGAGCCAGA | AGATGTTCTCAAACGCTTCG | 60 | Nygard et al., 2007 |
| IFNy | GCTCTGGGAAACTGAATGAC | TCTCTGGCCTTGGAACATAG | 60 | Meurens et al., 2009 |
| TNFalpha | CCAATGGCAGAGTGGGTATG | TGAAGAGGACCTGGGAGTAG | 62 | Meurens et al., 2009 |
| IL-2 | GCCATTGCTGCTGGATTTAC | CCCTCCAGAGCTTTGAGTTC | 63 | Meurens et al., 2009 |
| IL-6 | ATCAGGAGACCTGCTTGATG | TGGTGGCTTTGTCTGGATTC | 62 | Meurens et al., 2009 |
