# Supplementary material for: Neonatal Exposure to Amoxicillin Alters Long-Term Immune Response Despite Transient Effects on Gut-Microbiota in Piglets
Source: Front Immunol. 2019 Sep 4;10:2059. doi: 10.3389/fimmu.2019.02059 (PMC6737505; doi:10.3389/fimmu.2019.02059)
Supplement: Supplementary Table 3 — In vitro cytokine secretion from PHA stimulated PBMCs between AB and PL piglets on PND 21 and 491. 1Data present as mean ± pooled SEM. Data was analyzed using a Two-way ANOVA. AB, antibiotic; PL, placebo; PHA, phytohaemagglutinin; PBMC, peripheral blood mononuclear cell; PND, post-natal day; SEM, pooled standard error of the mean. PND 21: AB n = 7, PL n = 7; PND 49: AB n = 7, PL n = 8. [file Table_3.docx]

|  | **PND 21** | |  | **PND 49** | |  | **SEM** | ***P* values** | | |
| --- | --- | --- | --- | --- | --- | --- | --- | --- | --- | --- |
|  | **PL** | **AB** |  | **PL** | **AB** |  |  | (Treatment) | (Day) | **(**Day × Treatment) |
| **Cytokines (pg/mL)** |  |  |  |  |  |  |  |  |  |  |
| IL-2 | 187.7 | 225.8 |  | 163.3 | 170.3 |  | 24.8 | 0.547 | 0.353 | 0.898 |
| IL-6 | 646.0 | 420.1 |  | 553.5 | 400.6 |  | 48.6 | 0.347 | 0.101 | 0.283 |
| IL-10 | 316.8 | 533.6 |  | 492.4 | 439.0 |  | 47.6 | 0.401 | 0.675 | 0.170 |

**Supplementary Table 3.** *In vitro* cytokine secretion from PHA stimulated PBMCs between AB and PL piglets on PND 21 and 49^1^

^1^Data present as mean ± pooled SEM. Data was analyzed using a Two-way ANOVA. AB, antibiotic; PL, placebo; PHA, phytohaemagglutinin; PBMC, peripheral blood mononuclear cell; PND, post-natal day; SEM, pooled standard error of the mean. PND 21: AB n = 7, PL n = 7; PND 49: AB n = 7, PL n = 8.
