# Supplementary material for: Neonatal Exposure to Amoxicillin Alters Long-Term Immune Response Despite Transient Effects on Gut-Microbiota in Piglets
Source: Front Immunol. 2019 Sep 4;10:2059. doi: 10.3389/fimmu.2019.02059 (PMC6737505; doi:10.3389/fimmu.2019.02059)
Supplement: Supplementary Table 4 — Relative abundance of predominant fecal genera on PND 3 of AB and PL piglets1. 1Data presented as mean ± pooled SEM. Data was analyzed using a non-parametric Kruskall Wallis test. AB n = 20, PL n = 21. AB, antibiotic; PL, placebo; PND, post-natal day; SEM, pooled standard error of the mean; n/d, not detected. [file Table_4.DOCX]

**Supplementary Table 4.** Relative abundance of predominant fecal genera on PND 3 of AB and PL piglets^1^

|  | **AB** | **PL** | **SEM** | ***P* value** |
| --- | --- | --- | --- | --- |
| **Phyla** |  |  |  |  |
| *Actinobacteria* | 0.37 | 0.45 | 0.054 | 0.487 |
| *Bacteroidetes* | 39.90 | 39.32 | 2.467 | 0.908 |
| *Firmicutes* | 15.54 | 23.04 | 1.567 | 0.015 |
| *Fusobacteria* | 11.48 | 16.77 | 1.374 | 0.053 |
| *Proteobacteria* | 32.37 | 19.46 | 3.225 | 0.044 |
| *Verrucomicrobia* | 0.10 | 0.80 | 0.239 | 0.147 |
| **Genus** |  |  |  |  |
| **Actinobacteria** |  |  |  |  |
| g__*Arcanobacterium* | 0.10 | 0.09 | 0.029 | 0.898 |
| **Bacteroidetes** |  |  |  |  |
| g__*Butyricimonas* | 0.14 | 0.49 | 0.195 | 0.592 |
| g__*[Prevotella]* | 0.07 | 1.98 | 0.535 | 0.046 |
| g__*Bacteroides* | 37.04 | 34.29 | 2.276 | 0.525 |
| g__*Parabacteroides* | 1.84 | 1.06 | 0.267 | 0.152 |
| g__*Porphyromonas* | 0.71 | 0.04 | 0.224 | 0.090 |
| g__*Prevotella* | 0.02 | 1.26 | 0.523 | 0.138 |
| f__*Enterococcaceae*;Other | 0.51 | 0.28 | 0.111 | 0.333 |
| **Firmicutes** |  |  |  |  |
| g__*Lactobacillus* | 2.69 | 6.18 | 0.969 | 0.065 |
| g__*Streptococcus* | 0.54 | 1.04 | 0.252 | 0.425 |
| o__*Clostridiales*;f__;g__ | 0.62 | 1.08 | 0.203 | 0.286 |
| f__[*Mogibacteriaceae*];g__ | 0.04 | 0.15 | 0.040 | 0.179 |
| f__*Clostridiaceae*;g__ | 0.94 | 0.65 | 0.211 | 0.529 |
| g__*Clostridium* | 2.55 | 2.72 | 0.459 | 0.850 |
| f__*Lachnospiraceae*;g__ | 2.25 | 2.99 | 0.372 | 0.331 |
| g__[*Ruminococcus*] | 0.74 | 1.07 | 0.147 | 0.283 |
| g__*Coprococcus* | 0.03 | 0.11 | 0.022 | 0.090 |
| g__*Dorea* | 0.70 | 0.57 | 0.106 | 0.610 |
| g__*Roseburia* | 0.01 | 0.23 | 0.072 | 0.172 |
| f__*Peptostreptococcaceae*;g__ | 0.41 | 0.18 | 0.052 | 0.028 |
| g__*Peptostreptococcus* | 0.14 | 0.29 | 0.036 | 0.043 |
| f__*Ruminococcaceae*;g__ | 0.75 | 2.19 | 0.499 | 0.175 |
| g__*Oscillospira* | 0.41 | 0.60 | 0.125 | 0.475 |
| g__*Ruminococcus* | 0.29 | 0.41 | 0.062 | 0.337 |
| g__*Dialister* | 0.11 | 0.06 | 0.021 | 0.242 |
| g__*Mitsuokella* | 0.11 | 0.17 | 0.051 | 0.627 |
| g__*Phascolarctobacterium* | 0.12 | 0.06 | 0.033 | 0.370 |
| g__*Veillonella* | 0.43 | 0.82 | 0.093 | 0.027 |
| f__*Erysipelotrichaceae*;g__ | 0.09 | 0.18 | 0.037 | 0.236 |
| g__[*Eubacterium*] | 0.53 | 0.47 | 0.074 | 0.695 |
| **Fusobacteria** |  |  |  |  |
| g__*Fusobacterium* | 11.48 | 16.77 | 1.374 | 0.050 |
| **Proteobacteria** |  |  |  |  |
| g__*Sutterella* | 0.83 | 2.02 | 0.285 | 0.036 |
| g__*Bilophila* | 0.10 | 0.15 | 0.021 | 0.256 |
| g__*Desulfovibrio* | 0.33 | 0.54 | 0.066 | 0.134 |
| g__*Campylobacter* | 0.09 | 1.27 | 0.462 | 0.479 |
| f__*Enterobacteriaceae*;g__ | 29.3 | 14.3 | 3.300 | 0.027 |
| g__*Klebsiella* | 0.46 | 0.03 | 0.177 | 0.063 |
| g__*Actinobacillus* | 0.90 | 0.54 | 0.159 | 0.273 |
| g__*Aggregatibacter* | 0.18 | 0.41 | 0.068 | 0.100 |
| g__*Akkermansia* | 0.10 | 0.80 | 0.240 | 0.217 |
| Other | 1.38 | 1.52 | - | - |

^1^Data presented as mean ± pooled SEM. Data was analyzed using a non-parametric Kruskall Wallis test. AB n = 20, PL n = 21. AB, antibiotic; PL, placebo; PND, post-natal day; SEM, pooled standard error of the mean; n/d = not detected.
