# Supplementary material for: Neonatal Exposure to Amoxicillin Alters Long-Term Immune Response Despite Transient Effects on Gut-Microbiota in Piglets
Source: Front Immunol. 2019 Sep 4;10:2059. doi: 10.3389/fimmu.2019.02059 (PMC6737505; doi:10.3389/fimmu.2019.02059)
Supplement: Supplementary Table 5 — Relative abundance of predominant fecal phyla of AB an PL pigs on PND 3–491. 1Data presented as mean ± pooled SEM. Within a row, mean values without a common superscript differ (P < 0.05) or tend to differ (P ≤ 0.10). Data was analyzed using a non-parametric Kruskall Wallis test, with post-hoc pair-wise comparisons made using Dwass, Steel, Cirtchlow-Fliger multiple comparison procedure. PND 3 n = 41, PND 7 n = 38, PND 14 n = 39, PND 21 n = 39, PND 35 n = 42, PND 49 n = 39. AB, antibiotic; PL, placebo; PND, post-natal day; SEM, pooled standard error of the mean; n/d, not detected. [file Table_5.DOCX]

**Supplementary Table 5.** Relative abundance of predominant fecal phyla of AB an PL pigs on PND 3-49^1^

| **Phyla** | **PND 3** | **PND 7** | **PND 14** | **PND 21** | **PND 35** | **PND 49** | **SEM** | ***P* value** |
| --- | --- | --- | --- | --- | --- | --- | --- | --- |
| *Bacteroidetes* | 39.5^cd^ | 46.0^bc^ | 38.7^cd^ | 35.1^d^ | 54.2^ab^ | 59.2^a^ | 2.13 | < 0.001 |
| *Proteobacteria* | 25.7^ab^ | 18.3^bc^ | 27.7^a^ | 14.5^c^ | 3.50^d^ | 4.14^d^ | 2.16 | < 0.001 |
| *Firmicutes* | 19.4^e^ | 23.5^de^ | 25.5^cd^ | 43.9^a^ | 33.9^b^ | 30.7^bc^ | 1.52 | < 0.001 |
| *Fusobacteria* | 14.2^a^ | 7.78^b^ | 2.26^c^ | 0.73c | n/d | 0.01^c^ | 0.79 | < 0.001 |
| *Actinobateria* | 0.41^b^ | 2.0^a^ | 2.89^a^ | 1.94^a^ | 0.10^b^ | 0.16^b^ | 0.33 | < 0.001 |
| *Verrucomicrobia* | 0.50 | 2.02 | 1.75 | 1.45 | 1.13 | 0.01 | 0.67 | 0.222 |
| *Spirochaetes* | n/d | 0.28^c^ | 0.12^c^ | 0.96^bc^ | 4.28^a^ | 2.46^b^ | 0.39 | < 0.001 |
| Other | 0.19 | 0.21 | 0.99 | 1.50 | 2.93 | 3.27 | - | - |

^1^Data presented as mean ± pooled SEM. Within a row, mean values without a common superscript differ (*P* < 0.05) or tend to differ (*P* ≤ 0.10). Data was analyzed using a non-parametric Kruskall Wallis test, with post-hoc pair-wise comparisons made using Dwass, Steel, Cirtchlow-Fliger multiple comparison procedure. PND 3 n = 41, PND 7 n =38, PND 14 n = 39, PND 21 n = 39, PND 35 n = 42, PND 49 n = 39. AB, antibiotic; PL, placebo; PND, post-natal day; SEM, pooled standard error of the mean; n/d = not detected.
